# The Rational Use of Vulcanizable Rubber

**Published:** 1891-07

**Authors:** W. N. Murphy

**Affiliations:** Lagrange, Texas.


ARTICLE V.

### THE RATIONAL USE OF VULCANIZABLE RUBBER.

W. N. MURPHY, D. D. S., LAGRANGE, TEXAS.

[Read before the Texas Dental Association, at Waco, Texas, at its Annual Session, 1891.

Vulcanizable rubber, having become almost the universally accepted material as a base for artificial dentures, it behooves us to avail ourselves of the best methods possible in order to attain the highest results. In order to do this, it becomes necessary for us to study it from a scientific, as well as a practical point of view. But, however, much I may desire to go into the scientific details of it, the time allotted to this paper is too short; therefore I can only scan it briefly in order to deal more with the practical side of the question. Rubber becomes liquid at about 275 degrees Fahr., and a plate of pure rubber, with its coloring matter is then thrown out against the investment incasing it, as shown by figure No. 1, in such a way as to form a shield, or complete incasement, for the inner portion of the mass which is made up of rods of rubber extending from center to circumference, forming tubes filled with sulphureted hydrogen gas, or, in other words, the pressure of the gas forms the tubes. This gas escapes freely as long as the rubber remains in a liquid state. But, at about 300 degrees Fahr., the rubber begins to harden on the surface, and as the temperature is raised the hardness increases, penetrating deeper into the mass. When the rubber begins to harden on the surface the retention of gas begins and a slight expansion takes place. As the pressure of the gas increases which has a tendency to force the rubber to the circumference, leaving the tubes larger in the center and the rods smaller, as shown by figure No. 2. The larger or the thicker the mass of rubber, the greater the volume of gas and the larger the tubes, consequently the more porous the plate when the outer surface is removed in the finishing process. If the plate is very porous, which is the case in thick places around the alveolar ridge, when the surface is scraped off the rods will have a tendency to curl upon themselves, as shown by figure No. 3. This will draw the rubber away from the necks of the teeth and pins, sometimes making them loose on the plate, though this is not the only cause for this defect in dental plates. When the surface is removed, and this porosity exposed, it furnishes receptacles for the accumulation of effete gases which, I am sorry to say, are in almost every human mouth.

Then rubber having the following practical objections, viz: discoloration (especially pink), breakage, porosity, contraction or drawing away from the teeth, as designated by some, and incompatibility with the mucous membrane of the mouth, the question arises: How are we to impart to it that elasticity, toughness, lightness and strength coupled with the bright and translucent life-like appearance that should characterize every artificial denture, and at the same time restore or prevent a sunken in or contracted facial expression, and give the best results in the preparation of the food for alimentation? But in taking up the last prerequisite for a successful denture I shall have to digress to discuss the merits and demerits of plain teeth and gum secretions. Notwithstanding the almost endless variety of sizes, shades, etc., of gum sections furnished us by our progressive manufacturers, there are many, yea a large majority of the cases arising in the practice of a busy dentist in which it is impossible to give a proper accluding surface and maintain symmetry and beauty with anything but plain teeth. With the more improved forms of plain teeth, with their broader masticating surface, we are enabled to contort them into such position as to meet the demands of almost any conceivable case and to mislead the eye of the critic. Then in order to overcome, to a great extent, all and singular the foregoing objections, except that of incompatability, we will proceed to construct a full denture, supposing the mouth to be ready for a permanent set, although it is not essentially different for a temporary one. Take upper impression with plaster, using salt to hasten the settling and a few drops of cochineal in the water to color it. Take all lowers with modeling composition, never using it after it becomes old and tough. After making models boil them fifteen or twenty minutes in sterine and prepare wax forms for taking bite, which should be carefully done, so as to indicate the exact length for the teeth, then harden in cold water. Now place a thin layer of soft wax on one of them and put both in the mouth, putting the left hand on the forehead and the right on the back of the neck, force the patient's head back and at the same time directing to close the mouth and hold firm, which will insure a natural closure. The median line is then marked up and down, the position is then noted or marked for the canine teeth. Then, with the point of a small instrument, make a mark in the wax rim from one corner of the mouth to the other, equally dividing the lips. The case is then put in the articulator, and a strip of paper, about one-fourth of an inch wide, is pasted into the upper model with a drop of hot wax, one at each corner of the mouth and one in the center, and coming down to the mark indicating the dividing line for the lips. Plain teeth are then mounted accordingly and the wax gums carved up just as they are to be when the case is completed and ready for the mouth. And I wish to be understood by this that there is to be no scraping, filling or polishing to complete the case after it is vulcanized, except the little to be mentioned later on. Now, having carved up the wax gums to the proper form, thickness, etc., cut a pattern of No. 60 tinfoil to cover all of the lingual surface of the wax, which is to be well burnished down on that portion of the wax, which is to be only one thickness of pink, with no air chamber under it. The tin is then so trimmed and adjusted as to completely conform to the necks of the teeth. A strip, say one-fourth of an inch wider than the wax rim of the alveolar ridge, is then drawn round from heel to heel, and so completely and so nicely burnished down to the wax, and so accurately trimmed and adjusted to the festoon margin of the gums that no trace of wax that is to be replaced by rubber will be left exposed to the plaster investment; there should be no trace of wax left on the teeth. The upper edge or the excess in width of tinfoil is then cut into V-shape notches and the V's turned at right angles, then it is ready to invest right up to the upper edge of the wax, with the teeth down; the V's are then turned down into the soft investment of the first half, so as to prevent the tinfoil tearing down in flasking the case after it is packed. The wax is now washed out and the teeth, pins, and all parts are wiped off with chloroform so as to remove all traces of wax. The packing is then done so as to have just one thickness of polson pink rubber as a facing for that portion of the labial surface exposed to view when in the mouth, no traces of the red rubber being visible through the pink. No waste gates are to be used, consequently great care must be taken in the process of packing. Now run the vulcanizer up to 275 degrees and let it stand fifteen minutes, then take fifteen minutes to run up to 310, and then cool down slowly.—*Texas Dental Journal.*

